# Targeting miR-21 in spinal cord injuries: a game-changer?

**DOI:** 10.1186/s10020-022-00546-w

**Published:** 2022-09-23

**Authors:** Amir Mohammad Malvandi, Seyed Hamidreza Rastegar-moghaddam, Saeede Ebrahimzadeh-Bideskan, Giovanni Lombardi, Alireza Ebrahimzadeh-Bideskan, Abbas Mohammadipour

**Affiliations:** 1 Laboratory of Experimental Biochemistry and Molecular Biology, IRCCS Istituto Ortopedico Galeazzi, Via Cristina Belgioioso 173, 20157 Milan, Italy; 2 Student Research Committee, Mashhad University of Medical Sciences, Mashhad, Iran; 3 Department of Anatomy and Cell Biology, Faculty of Medicine, School of Medicine, Mashhad University of Medical Sciences, Azadi Sq, Vakilabad Blvd, Mashhad, Iran; 4 Rehabilitation Division, Ghaem Hospital, Mashhad University of Medical Sciences, Mashhad, Iran; 5 Department of Athletics, Strength and Conditioning, Poznań University of Physical Education, Poznań, Poland; 6 Applied Biomedical Research Center, School of Medicine, Mashhad University of Medical Sciences, Mashhad, Iran

**Keywords:** Spinal cord injury, MicroRNA-21, Anti-inflammatory, Anti-apoptotic, Angiogenesis, Neural stem cells

## Abstract

Spinal cord injury (SCI) is a devastating neurological state causing physical disability, psychological stress and financial burden. SCI global rate is estimated between 250,000 and 500,000 individuals every year, of which 60% of victims are young, healthy males between 15 and 35 years. A variety of pathological conditions such as neuroinflammation, mitochondrial dysfunction, apoptosis, glial scar formation, blood-spinal cord barrier disruption, and angiogenesis disruption occur after SCI leading to a limitation in recovery. MicroRNAs (miRs) are endogenous and non-coding RNAs consisting of 22 nucleotides that regulate 60% of all human genes and involve several normal physiological processes and pathological conditions. miR-21 is among the most highly expressed miRs and its expression has been shown to increase one day after SCI and this elevation is sustained up to 28 days after injury. Overexpression of miR-21 exerts many protective effects against SCI by inhibiting neuroinflammation, improving blood-spinal cord barrier function, regulating angiogenesis, and controlling glial scar formation. It also exhibits anti-apoptotic effects in SCI by down-regulating the expression of PTEN, Spry2, and PDCD4. This review provides a novel therapeutic perspective for miR-21 in SCI.

## Background

Spinal cord injury (SCI) is a destructive neurological state causing dysfunction in the primary motor, sensory and autonomic neural system affecting prevalently (60%) 15–35 years old healthy males (van Den Hauwe et al. 2020). It is estimated to involve 250,000–500,000 individuals every year worldwide (Anjum et al. 2020), and the costs for each patient can arrive at 3 million dollars in a whole lifetime perspective (Katoh et al. 2019). So far, there is no treatment available due to SCI complex physiopathology. A better understanding of SCI at the molecular level can reveal recovery mechanisms and discover some biological therapeutics potentially promising to confront SCI.

MicroRNAs (miRs) are small nucleic acids involved in post-transcriptional regulatory mechanisms (Rastegar-Moghaddam et al. 2022a). These biomarkers are expressed in all body tissues with a higher level in the central nervous system (CNS) (Rastegar-Moghaddam et al. 2022b). Around 70% of known miRs are expressed in CNS (Kou et al. 2020). miRs play critical roles in several cellular and molecular mechanisms, including angiogenesis, energy-providing, neuronal differentiation, maturation, and survival (Rastegar-Moghaddam et al. 2022b; Tonacci et al. 2019).

miR-21 is one of the most expressed miRs. It contains 22 nucleotides encoded by a sequence located within the Vacuole Membrane Protein-1 (VMP-1) gene on chromosome 17 (Jenike and Halushka 2021; Surina et al. 2021). It is expressed widely in various body tissues, including the spinal cord and its expression is altered following SCI (Liu et al. 2018; Chung et al. 2020). Several studies demonstrated that miR-21 has many protective roles against SCI by decreasing apoptosis and increasing neuron survival and can be considered a potential potent therapeutics (Kang et al. 2019; Zhang et al. 2019; Lv et al. 2020; Wang et al. 2021).

### Pathophysiology of SCI

Following SCI, neurons and glial cells die due to various factors, including ionic imbalance and glutamate excitotoxicity, proinflammatory cytokines release, free radical production, ATP depletion, and apoptosis (Alizadeh et al. 2019; Samandari et al. 2019; Anjum et al. 2020). A hallmark of SCI is vascular and angiogenesis disruption, resulting in reduced oxygen delivery and mitochondrial dysfunction. Subsequently, ATP depletion, calcium overload, excitotoxicity, and oxidative stress exacerbate injury (Vasiliadis et al. 2014; Scholpa et al. 2017; Anjum et al. 2020). Neurons depend highly on ATP for ion exchange and maintaining electrochemical and energy homeostasis. Because energy demand in neurons is several times greater than in other cells, they are more vulnerable to ATP depletion (Mohammadipour et al. 2020; Malvandi et al. 2021). Inflammation is another phenomenon associated with SCI, which increases neural damage by causing edema, apoptosis, and reactive gliosis (Rong et al. 2019; Anjum et al. 2020). The local expression of interleukin (IL)-1, IL-6, IL-8, and tumor necrosis factor (TNF)-α increases following SCI (Rong et al. 2019; Slota and Booth 2019; Lv et al. 2020). In addition, the augment in the expression of apoptotic proteins such as Bax, caspase-3, and caspase-9, phosphatase and tensin homolog (PTEN), and programmed cell death protein 4 (PDCD4) increase neuronal death (Kang et al. 2019; Hausott and Klimaschewski 2019).

### Anti-neuroinflammatory effects

Although miR-21 has some inflammatory functions, it appears as predominantly anti-inflammatory miR in the nervous system and could effectively modulate neuroinflammation (Gaudet et al. 2018; Slota and Booth 2019). Indeed, suppression of miR-21 promotes IL-1β, IL-6, TNF-α (Table 1), and receptor activator of nuclear factor kappa-Β ligand (RANKL), leading to severe inflammation (Zhou et al. 2018). Conversely, miR-21 declines inflammatory factors such as IL-1β, IL-6, IL-8, TNF-α, and endothelial nitric oxide synthase (eNOS), and it enhances the anti-inflammatory cytokine IL-10 (Slota and Booth 2019; Lv et al. 2020).

**Table 1:** The biological effects and main related mechanisms of miR-21

Biological effects	Main mechanisms	References
Anti-inflammation	• Reduces IL-1β, IL-6, IL-8, TNF-α, eNOS • Downregulates CCL3	Lv et al. (2020) Liu et al. (2020)
Anti-apoptotic	• Reduces Bax/Bcl-2, and Caspase-3 and Caspase-9, and PTEN protein expressions • Reduces PDCD4	Hu et.al. (2013) Zhang et al. (2019)
Anti-glial scar formation	• Modulates astrocytes’ secretion, proliferation, and apoptosis • Modulates PI3K/Akt/mTOR • Reduces astrocytes hypertrophy in the SCI	Liu et al. (2018) Liu et al. (2018) Bhalala et al. (2012)
Angiogenesis modulation	• Inhibits TIMP3 and promotes MMP2 and MMP9 • Promotes expression of Ang-1, Tie-2, and VEGF •Increases MMP-13 and p-ERK1/2 • Promotes the survival, migration and tube formation of endothelial cells	Hu et al. (2016) Ge et al. (2014) Ma et al. (2020) Hu et al. (2016)
Neuroregeneration modulation	• Promotes neural differentiation of NSPCs • Enhances the expression of cyclin D1 in NSPCs • Activates AKT/GSK-3β signaling Pathway • Modulates Wnt/β-catenin signaling pathway	Gao et al. (2016) Song et al. (2021) Gao et al. (2016) Zhang et al. (2018)

**Fig. 1:**
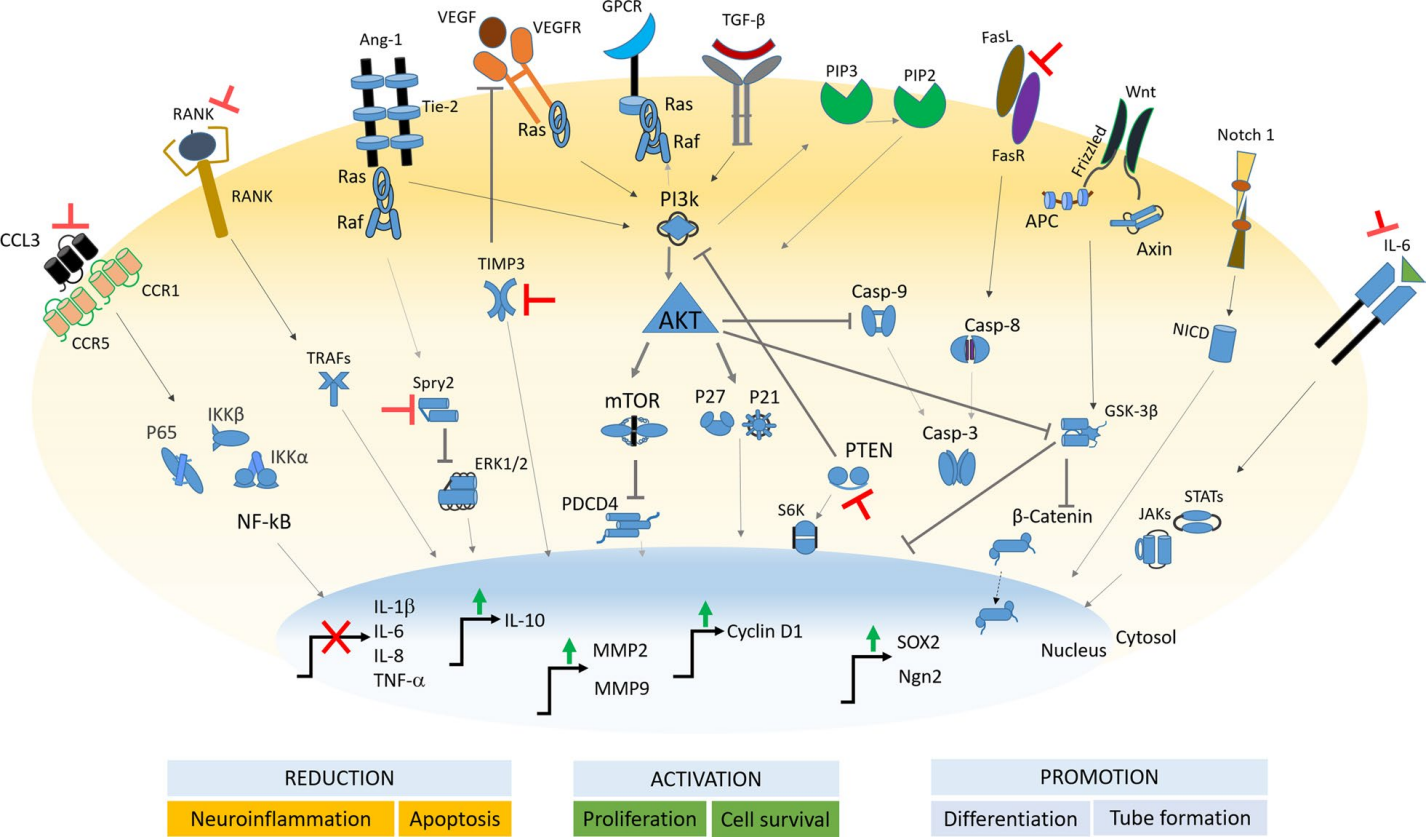
miR-21 global molecular mechanism of action leading to functional effects on neural cells’ status and function. ⊥ Represents inhibition, → shows induction/promotion of activity

An experiment executed in a rat model of SCI showed that miR-21 overexpression could reduce the expression of IL-1β, IL-6, IL-8, and TNF-α (Lv et al. 2020). In the neonatal rat ischemia model, miR-21 showed repressor activity on proinflammatory C–C motif chemokine ligand 3 (CCL3), favoring neuroprotection (Liu et al. 2020). CCL3 and its receptors are induced after SCI and contribute to progressive tissue damage and functional impairment during secondary injury (Pelisch et al. 2020). CCL3 also activates the nuclear factor kappa B (NF-κB) signaling pathway (Fig. 1), which is a hallmark of inflammation (Mohammadipour et al. 2021). miR-21 negatively regulates CCL3, repressing -in turn- IKKα/β and p65 phosphorylation, disrupting the NF-κB signaling pathway (Liu et al. 2020). miR-21 also suppresses different target components of the toll-like receptor (TLR)/MyD88/NF-κB and JAK-STAT pathways (Slota and Booth 2019).

Therefore, miR-21 overexpression seems to play a fundamental role in decreasing secondary injury after SCI by reducing the expression of inflammatory factors and suppressing inflammation.

### Glial scar formation

miR-21 has a protective effect on SCI by controlling glial scar formation. Glial scar is believed to play a dual role in the pathological process of SCI (Yang et al. 2020). Although glial scar has some protective roles, they also have many detrimental effects after SCI. Glial scar is the most crucial inhibitor factor to neuroregeneration after SCI (Leal-Filho et al. 2011) and is a significant limitation in improving outcomes (Bhalala et al. 2012). In the uninjured spinal cord, miR-21 expression is neither silenced nor overexpressed in astrocytes, which indicates that this miR is not essential for maintaining astrocyte homeostasis. However, its expression rate increases sharply after SCI and reaches its maximum five weeks after injury (Bhalala et al. 2012). miR-21 has been found to act in astrocytes to control their functions and regulate the astrocytic size and glial scar formation after SCI (Table 1), interacting with bone morphogenetic protein (BMP) and JAK-STAT signaling pathways (Bhalala et al. 2012). This miR modulates astrocytes’ secretion, proliferation, and apoptosis (Fig. 1) to promote recovery through transforming growth factor (TGF)-β-mediated targeting of the phosphoinositide-3-kinase (PI3K)/Akt/mammalian target of rapamycin (mTOR) pathway (Liu et al. 2018). Overexpression of miR-21 reduces astrocyte hypertrophy in the traumatic SCI, whereas conversely, suppression of this miR induces astrocyte hypertrophy (Bhalala et al. 2012; Su et al. 2019).

### Angiogenesis

Immediately after SCI, damage to spinal cord microvascular endothelial cells (SCMECs) occurs and not only disrupts the blood-spinal cord barrier (BSCB) but also results in disrupted angiogenesis and reduced blood supply (Zhong et al. 2020; Jin et al. 2021). Angiogenesis is necessary for axonal regeneration by facilitating tissue remodeling and survival. Although due to endogenous angiogenesis, blood vessel density transiently increases within two weeks after SCI but it is insufficient (Yao et al. 2021). Vascular endothelial growth factor (VEGF) and metalloproteinases (MMPs), especially MMP-2, are among the pro-angiogenic mediators and play fundamental roles in preserving vascular integrity, regulating basal muscle capillarization, and microvascular remodeling (Vasiliadis et al. 2014).

miR-21 is a potential pro-angiogenic factor and it is reported that decreased level of this miR inhibits angiogenesis in a rat model of SCI (Hu et al. 2016). Overexpression of miR-21 promotes the expression of angiogenesis-related molecules, including angiopoietin-1 (Ang-1), Tie-2 (receptor of Ang-1), and vascular endothelial growth factor (VEGF) (Fig. 1) in the injured neural tissues (Ge et al. 2014). In addition, this miR promotes angiogenesis by increasing the expression of MMP-13 and p-ERK1/2 (Ma et al. 2020). Overexpression of miR-21 also promotes the survival, migration and tube formation of endothelial cells by inhibiting tissue inhibitor of metalloproteinase-3 (TIMP3) expression and promoting MMP2 and MMP9 expression (Hu et al. 2016) (Table 1).

### Neuroregeneration

Neural stem/progenitor cells (NSPCs) are essential for nerve regeneration after SCI (Wang et al. 2018). The main issue related to NSPCs is their poor proliferation rate and low differentiation efficiency into neurons, and if their proliferation and differentiation are promoted, it can have beneficial effects on the treatment of SCI. Besides endogenous NSPCs, the therapeutic effect of stem cell transplantation in SCI has been considered in recent studies, and it has been shown that miR-21-containing stem cell-derived exosomes promote the protective effects of stem cell transplantation against SCI, whereas miR-21 deficiency in stem cells did not exert such benefits (Ji et al. 2019). miR-21 overexpression has been found to promote neural differentiation of neural stem/precursor cells (NSPCs) (Gao et al. 2016). Overexpression of this miR enhances the expression of cyclin D1 in NSPCs (Fig. 1), a protein that has a role in the differentiation and survival of NSPCs (Song et al. 2021). A previous study showed that cyclin D1 expression is enhanced by Notch1, which promotes spinal NSPCs proliferation (Wang et al. 2018). Overexpression of this miR also positively regulates proliferation and neural differentiation of NSPCs by activating protein kinase B (AKT) and glycogen synthase kinase-3 beta (GSK-3β) signaling pathways.

Conversely, knocking down miR-21 reduces neural differentiation in NSPCs by preventing cyclin D1 expression and blocking AKT/GSK-3β (Table 1) (Gao et al. 2016). In addition to mentioned pathways, the Wnt/β-catenin pathway has exhibited an important role in regulating NSPCs fate and activation of this pathway has been demonstrated to promote NSPCs proliferation and differentiation (Zhang et al. 2018). It has been found that miR-21 can enhance the proliferation of neural stem cells and their differentiation into neurons and reduce stem cells’ differentiation into astrocytes via the Wnt/β-catenin signaling pathway (Zhang et al. 2018).

### The exercise and miR-21 after SCI

Exercise can be considered a non-invasive therapy for SCI due to its benefits for stabilizing rhythmic firing patterns of spinal motoneurons, maintaining the muscle mass of paralyzed limbs (Ning et al. 2014) and possibly bone mass (Sutor et al.^2022^) to improve functional recovery. Several studies have shown the beneficial effects of physical activity on SCI (Vasiliadis et al. 2014; Ying et al. 2021; Nash et al. 2022). Physical activity after SCI reduces inflammation (Donia et al. 2019), neuronal and glial apoptosis (Jung et al. 2014), and increases angiogenesis (Vasiliadis et al. 2014) and cell survival (Li et al. 2020), leading to improved recovery. Short-time physical activity also led to a significant increase in miR-21 expression, at the spinal cord level, after SCI (Ning et al. 2014). Li et al. recently showed that exercise after SCI increases miR-21 expression and decreases PDCD4 levels, leading to reduced apoptotic cell number (Li et al. 2020). Since miR-21 reduces inflammation and apoptosis and increases cell survival and angiogenesis after SCI, exercise can improve recovery by overexpressing this miR.

Exercising, regardless of the type, enhances the expression, and therefore the circulating levels, of miR-21 (Horak et al. 2018) and particularly the fraction associated with extracellular vesicles (Siqueira et al. 2021). However, there is little knowledge about the effect of exercise on the expression of this miR in the nervous system. There are reports about the exercise-induced expression of miR-21 in the endothelial compartment with an intensity- (Wahl et al. 2016) and volume-dependent manner (Kilian et al. 2016).

Exercise after SCI alters gene expression leading to increased spinal cord plasticity and recovery of spinal reflexes (Mendell et al. 2001; Ying et al. 2005; Côté et al. 2011), and passive hindlimb exercise after SCI was shown to attenuate the SCI-induced increase in miR-199a-3p, a negative regulator of miR-21, with concurrent upregulation of miR-21 (Liu et al. 2012). The dynamic equilibrium between miR-21 and miR-199a-3p is responsible for the regulation of mTOR and PTEN (Hu et al. 2013), which in turn has been shown to drive axon growth in vitro and in vivo (Kar et al. 2021). While the literature is poor in this field, there are reports about the exercise-dependent miR-21-related effects on traumatic brain injury (TBI). As miR-21 is involved in the signaling pathways of inflammation, neuronal apoptosis, reactive gliosis, disruption of the blood–brain barrier, and angiogenesis in TBI (i.e., it is considered a marker and therapeutic target in TBI (Martinez and Pepolow 2017)), its induction by exercise, in the nervous system, can improve the recovery process (Ji et al. 2018). Further, in mice models of TBI, spontaneous wheel running enhanced hippocampal expression of miR-21 is associated with improved recovery (Bao et al. 2014).

Further, miR-21 is expressed by the skeletal muscle during exercise (D’Souza et al. 2018). Muscle-derived miR-21 may act at the neuromuscular plaque, exerting effects in motoneurons and backward at the spinal cord level.

## Conclusions and future perspectives

Paralleling the evidence can reveal the protective potential for miR-21 against the burden of SCI through suppressing the inflammatory milieu in neural cells, apoptosis inhibition, improving angiogenesis, and synapsis protection (Fig. 1). It seems that miR-21 activates in response to the injury but declined in the following. The discovery of regulatory elements effective on miR-21 seems crucial and requires more effort.

SCI usually occurs accidentally, and there is an acute trauma period, potentially a crucial time to manage the pathology in a better condition. Current understanding showed favorable effects of miR-21 overexpression in a preclinical setting. Considering recent successful RNA-based therapeutics worldwide, it is worth exploring clinically. In this context, RNA-containing particles can be delivered using a direct administration approach.

The role of miR-21 in axon regeneration is unknown and should be more explicit to be helpful for a (pre)clinical investigation; however, the use of miR-21 as a neuroprotective along with other factors is suggested, for instance, inside a nanostructured scaffold can be mounted on the injury site-during the acute phase- to cure the consequences of the incident.

Notably, exercise, a therapeutic strategy commonly applied in the rehabilitation path of SCI patients, affects miR-21 expression at the nervous system levels. Therefore, it would be possible to enhance this effect by optimizing the rehabilitation program.

Last but not least, miR-21 is a crucial regulator of inflammatory conditions favoring metabolic resolution of the inflammatory milieu in the neural context. However, the effector target(s) and the microenvironment where miR-21 will be involved can revert the goal. The preclinical studies addressing efficacy and safety will essentially answer this doubt.

## Data Availability

All data generated or analyzed during this study are included in this published article.

## Abbreviations

ACT: Activating protein kinase B
Ang-1: Angiopoietin-1
BMP: Bone morphogenetic protein
CCL3: C–C motif chemokine ligand 3
CNS: Central nervous system
eNOS: Endothelial nitric oxide synthase
GSK-3β: Glycogen synthase kinase-3 beta
IL: Interleukin
miR: MicroRNA
MMPs: Metalloproteinases
mTOR: Mammalian target of rapamycin
NF-κB: Nuclear factor kappa B
NSPCs: Neural stem/progenitor cells
PDCD4: Programmed cell death protein 4
PI3K: Phosphoinositide-3-kinase
PTEN: Phosphatase and tensin homolog
RANKL: Receptor activator of nuclear factor kappa-Β ligand
SCI: Spinal cord injury
SCMECs: Spinal cord microvascular endothelial cells
TBI: Traumatic brain injury
TGF: Transforming growth factor
TIMP3: Tissue inhibitor of metalloproteinase-3
TLR: Toll-like receptor
TNF: Tumor necrosis factor
VEGF: Vascular endothelial growth factor
VMP-1: Vacuole membrane protein-1
